# ATARI: A Graph Convolutional Neural Network Approach for Performance Prediction in Next-Generation WLANs

**DOI:** 10.3390/s21134321

**Published:** 2021-06-24

**Authors:** Paola Soto, Miguel Camelo, Kevin Mets, Francesc Wilhelmi, David Góez, Luis A. Fletscher, Natalia Gaviria, Peter Hellinckx, Juan F. Botero, Steven Latré

**Affiliations:** 1 Department of Computer Science, University of Antwerp—imec, 2000 Antwerp, Belgium; miguel.camelo@uantwerpen.be (M.C.); kevin.mets@uantwerpen.be (K.M.); peter.hellinckx@uantwerpen.be (P.H.); steven.latre@uantwerpen.be (S.L.); 2 Department of Telecommunications Engineering, Universidad de Antioquia, Medellín 050010, Colombia; david.goez@udea.edu.co (D.G.); luis.fletscher@udea.edu.co (L.A.F.); natalia.gaviria@udea.edu.co (N.G.); juanf.botero@udea.edu.co (J.F.B.); 3 Centre Tecnològic de Telecomunicacions de Catalunya, 08860 Castelldefels, Spain; fwilhelmi@cttc.cat

**Keywords:** channel bonding, graph neural network, machine learning, performance prediction, WLANs

## Abstract

IEEE 802.11 (Wi-Fi) is one of the technologies that provides high performance with a high density of connected devices to support emerging demanding services, such as virtual and augmented reality. However, in highly dense deployments, Wi-Fi performance is severely affected by interference. This problem is even worse in new standards, such as 802.11n/ac, where new features such as Channel Bonding (CB) are introduced to increase network capacity but at the cost of using wider spectrum channels. Finding the best channel assignment in dense deployments under dynamic environments with CB is challenging, given its combinatorial nature. Therefore, the use of analytical or system models to predict Wi-Fi performance after potential changes (e.g., dynamic channel selection with CB, and the deployment of new devices) are not suitable, due to either low accuracy or high computational cost. This paper presents a novel, data-driven approach to speed up this process, using a Graph Neural Network (GNN) model that exploits the information carried in the deployment’s topology and the intricate wireless interactions to predict Wi-Fi performance with high accuracy. The evaluation results show that preserving the graph structure in the learning process obtains a 64% increase versus a naive approach, and around 55% compared to other Machine Learning (ML) approaches when using all training features.

## 1. Introduction

The global number of Wi-Fi hotspots (including homespots) will increase four-fold by 2023, providing connectivity to 16.2 billion devices with approximately 3.6 networked devices per capita [1]. Additionally, Machine-to-Machine (M2M) communications will account for 50% of those networked devices, which represents an increase of 17%, compared to 2018. This means that, not only in residential settings, Wi-Fi will be one of the preferred technologies that gives internet access to a myriad of networked devices to support bandwidth-hungry services, such as augmented and virtual reality, online gaming and video streaming. Moreover, recent Long-Term Evolution (LTE) versions propose to use unlicensed bands (<5 GHz) as an traffic offloading solution to cope with the increasing mobile traffic demand.

As network-oriented applications evolve toward increasingly stringent Quality of Service (QoS)/Quality of Experience (QoE) requirements, Wi-Fi amendments have defined new strategies that improve the offered bandwidth to users. In this regard, future Wi-Fi generations are proposing a set of features from which Channel Bonding (CB) is highlighted. CB is a technique introduced in 802.11n that enhances the channel capacity by bonding a maximum of two primary channels in a given transmission. Newer Wi-Fi versions allow even more primary channels to be bonded to increase bandwidth [2].

Notwithstanding, the novel mechanisms for next-generation Wi-Fi deployments, addressing the demands of highly dense scenarios, adds more stress to the already scarce spectrum, as more devices will fight for medium access. Even if the LTE offloaded traffic is not taken into account, CB in crowded Wi-Fi scenarios might be counterproductive, as end-user Stations (STAs) might overlap. As a result, the STAs may experience a high number of collisions and suffer excessive starvation [3]. This situation can cause drastic performance deterioration and increase the packet error rate. Therefore, intelligent spectrum access strategies are of the utmost importance to accomplish QoS requirements of all connected devices.

For instance, dynamic configurations of CB (Dynamic Channel Bonding (DCB)), where the number of bonded channels is adaptively changed depending on network conditions, are recommended to increase the throughput on the one hand (when the channel is not congested), and to alleviate the harmful effects of interference on the other hand (when congestion is high) [4]. However, it is not always clear for an Access Point (AP) which configuration to apply in a given situation of DCB. For that, we argue that performance (e.g., throughput) prediction/estimation could help in optimizing wireless networks by assessing the feasibility of potential system changes. Particularly in CB, performance prediction allows Wireless Local Area Networks (WLANs) to select channel configurations that minimize the inter-Basic Service Set (BSS) interference, optimizing the spectrum usage.

To predict its performance, controllers or APs in a WLAN can use network models to assess how positively or negatively a given decision will impact the performance of the WLAN. Typically, Markov models [5] and mathematical models [6] have been used to characterize WLANs. However, given the increasing heterogeneity in network services and functionalities that are developed to accommodate the extreme imposed requirements, traditional mathematical models do not hold. On the one hand, the Markov chain states increase exponentially with the number of considered devices and their configurations. On the other hand, mathematical approaches rely on simplifying assumptions in order to keep tractability. Moreover, new services and technologies alter the traffic patterns, forcing prediction models to be continuously updated. Therefore, new models are needed that adapt to varying network conditions and environments.

ML approaches have taken advantage of the myriad of data sources to solve complex problems where analytical models are intractable. Such an abundance of data makes ML powerful for different aspects of network optimization, as they avoid making *a priori* assumptions, typically found on analytical models. Although ML takes advantage of rich data sets, that may not be the case for wireless networks, where it is difficult to obtain enough data. Data extracted from real deployments are often limited by privacy issues (e.g., proprietary data from a network operator) or sparsity (e.g., deployments with a couple of transmitters and receivers). On the contrary, synthetic data can be easily generated where multiple deployments with a high number of transmitting devices and different configurations can be evaluated through simulations [7]. These synthetic data can train accurate ML models that adapt well to real and changing situations.

To predict the throughput in IEEE 802.11 WLANs that support DCB, we propose in this paper a novel approach, using a GNN architecture. GNNs are Neural Networks (NNs) that operate on data represented as graphs and exploit the relationship among nodes embedded in the graph’s topology. Additionally, we propose two Deep Learning (DL)-based models and an ML-based model that serve as a comparison for our GNN approach. We train all models with data generated by Komondor [8], a simulator for next-generation, high-density WLANs. The generated data use different random parameters, including channel allocation, location of nodes, and number of STAs per AP. Our trained GNN model can be used later to optimize the planning phase of a given deployment or improve the performance during a WLAN operation. Our contributions can be summarized as follows:

- We propose a GNN model that predicts the achieved throughput in highly dense IEEE 802.11 WLANs using CB. To the best of our knowledge, this is the first time GNNs are applied to this problem.
- We compare our approach with recent state-of-the-art DL and ML approaches and discuss how different features impact the prediction accuracy. Based on the available features, a given model can be optimally selected.
- Our proposal accurately predicts the throughput generating a model that can be employed in future intelligent decision frameworks for CB, similar to [9].

The remainder of this paper is organized as follows. An overview of the related work is presented in Section 2. Section 3 discusses the necessity of learning models in wireless environments. In Section 4, we present the proposed GNN model for throughput prediction in high-density WLANs capable of Channel Bonding. In Section 5, we present several ML approaches that serve as a comparison baseline to the GNN model. Section 7 shows the performance and the numerical comparison of the models. We discuss the approach in Section 8 and conclude the paper in Section 9.

## 2. Related Work

Several works have shown that using the same set of bonded channels in each transmission might be counterproductive, as neighboring nodes might interfere and prevent other nodes from transmitting in a given channel (or set of channels) [3]. These works also showed that the system performance could improve if dynamic CB policies can be applied based on current spectrum usage.

Under dynamic CB, the node will transmit over the most extensive contiguous set of channels sensed to be idle right before transmission. In that way, a node can adopt several channel configurations, depending on how many channels are free on a per-packet basis. An evaluation of different channel configurations is presented in [10]. Through simulations, the authors found that CB does not represent a significant increase in the overall throughput when considering a high amount of contending nodes. Instead, better performance is achieved when transmitting over single channels.

CB benefits from throughput prediction models, given that different channel configurations can be evaluated before being applied. The best configuration can be selected to increase the overall WLAN performance. Typically, Markov chains have been used as throughput prediction models. For instance, a continuous-time Markov-chain was proposed in [11] to evaluate the performance in fully overlapping deployments. Using this Markov chain, the authors could calculate the throughput achieved by an AP and its associated STAs and explain some fundamental properties in dynamic CB, such as their sensitivity to the backoff and transmission time distributions. Similarly, in [12], the authors considered partially overlapping deployments and proposed two more CB policies, including a probabilistic selection of channels. The obtained results showed that adaptive policies are needed that benefit from the information they can gather from the medium.

Learning models were also proposed for Wi-Fi performance prediction. In [13,14], classical ML techniques (e.g., shallow Feed Forward Neural Network (FNN), Support Vector Machine (SVM), Random Forest (RF), Gradient Boost (GB), among others) are used to predict the throughput in Wi-Fi networks in varying environmental and network conditions. Authors clearly state the importance of the set of input features in ML models, as in [13], their best results were obtained with an FNN at the expense of a linearly increasing feature set and computation time with the number of STAs in the network; in [14], the authors found that using features such as Received Signal Strength Indicator (RSSI) are only meaningful for prediction in coarse time-scales (e.g., typically seconds).

Different CB policies can be evaluated through Reinforcement Learning (RL), where APs learn what channels they need to bond depending on the expected throughput. Therefore, RL approaches need to evaluate the impact of the chosen configuration. For instance, in [15], based on a simple interference model, the authors proposed a Deep Q-Network (DQN) that learns how many channels need to be bonded to satisfy future demands. Here, the authors assumed that different WLANs are connected to a central controller, allowing information exchange. The results showed that the algorithm learns to avoid interference, given that the most-used channel configurations were the ones where most neighboring APs did not overlap with each other.

As it can be seen, different solutions have been proposed for modeling Wi-Fi performance. Most of them are mathematical models based on simple assumptions that lower their complexity and do not fully capture the wireless interactions. Some learning models have also been proposed for the same purpose and clearly state the importance of the input features in the prediction accuracy. The key differences between our work and previous works are summarized as follows:

- Instead of defining an analytical model that does not scale when the number of nodes in the environment increases, we propose several ML approaches that learn from data. The proposed approaches take care of the complex task of defining wireless interactions.
- Unlike [13,14], we focus on highly dense scenarios, where a variable number of nodes are sparse over a given area. Our scenarios range from 40 to 240 nodes per deployment in six different configurations of CB. We show these scenarios in Section 6.
- Unlike [15], our approach does not define the best CB policy that a WLAN needs to apply to maximize its reward. Our goal is that the ML model learns how the wireless interactions affect the throughput of a given WLAN without any explicit assumptions, such as user demand or user mobility patterns. This approach can later be used as a function approximator in such RL approaches.

## 3. Motivation for Learning Models in Next-Generation WLANs

In this paper, we consider a highly dense wireless environment, where multiple APs and STAs transmit using Wi-Fi with CB support. CB is one of the features [16] introduced in the newest amendments of Wi-Fi [2,17] to cope with the stringent demands from next-generation services. For instance, Wi-Fi can be used for content delivery in a shopping mall where several customers are connected. Consequently, a BSS consists of *M* nodes, one AP, and M−1 STAs (customers) associated with that AP. Multiple APs have to be deployed to provide connectivity to all users around the shopping mall. We also assume that not all the nodes are within others’ carrier-sensing range, causing well-known problems, such as hidden or exposed nodes.

**Figure 1:**
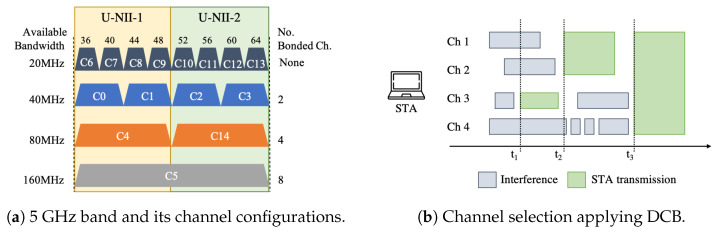
CB problem’s complexity is exacerbated by its combinatorial nature and the applied transmission policy.

When 802.11n/ac/ax APs use the lower part of the 5 GHz band (i.e., the U-NII-1 and U-NII-2 bands), there is a total of eight non-overlapping channels of 20 MHz bandwidth as shown in Figure 1a. In CB multiple adjacent channels can be bonded to create a higher bandwidth channel to potentially increase the throughput of a given transmission. The total bandwidth of CB has grown from 40 MHz (802.11n) to 160 MHz (802.11ac/ax) and it is expected to support up to 320 MHz (802.11be, using the 6 GHz band).

Under the constraint to bond only adjacent channels, the number of available configurations in a small portion of spectrum is 16 (8 × 20 MHz, 4 × 40 MHz, 2 × 80 MHz and 1 × 160 MHz). With the introduction of novel bonding techniques using Orthogonal Frequency Multiple Access (OFDMA), non-contiguous channels can be bonded, increasing the number of available configurations. Then, every AP is assigned with a contiguous subset of channels from *F* channels of equal bandwidth, according to a transmission policy. Such transmission policies can be static, dynamic or probabilistic. In Static Channel Bonding (SCB), the same subset of channels is selected in a per-packet basis transmission. In DCB, a variable subset is selected according to the available spectrum, as shown in Figure 1b. Finally, in probabilistic CB, a configuration is chosen with a given probability [12].

In Figure 1b, we assume a transmitting STA in which DCB can be applied over channels 1–4, using configuration C4 or C14 from Figure 1a. At time $t_{1}$, the STA senses that only channel 3 is available for transmission, using the Carrier-Sense Multiple Access with Collision Avoidance (CSMA/CA) procedure. Similarly, at $t_{2}$, channels 1 and 2 are sensed to be free at the moment of transmission and the two are bonded consequently. Finally, at $t_{3}$ the STA is able to transmit over the bonded channels 1–4. Note that, if a SCB policy is applied, the STA is able to transmit only at $t_{3}$, while if a probabilistic policy is used, any combination of channels can be selected in $t_{2}$ and $t_{3}$.

Hence, if the network conditions allow it, each AP uses the largest subset of available transmission channels allowed by its policy and depending on the wireless environment. The more channels it can bond, the higher the degree of freedom in selecting channel configurations. In this way, the AP tries to increase the overall throughput. Given the high number of BSS deployed in crowded deployments and the combinatorial nature of CB, it is not always trivial to select the right channel configuration.

Consequently, a careful selection of the transmission channels has to be done since selecting the widest subset of available channels can be harmful in the overall long-term throughput and fairness among WLANs [12]. Next-generation WLANs will be able to assess the impact of a given configuration before every transmission takes place through performance prediction models. In dense environments, multiple interactions among devices occur in a matter of microseconds. These interactions vary on a per-packet basis, making each transmission unique. Moreover, the wireless channel capacity where that transmission occurs depends on the channel effects, such as noise and interference and the number of bonded channels. Therefore, figures, such as Signal-to-Interference plus Noise Ratio (SINR), RSSI, bandwidth, Modulation and Coding Scheme (MCS), and the amount of time a node can transmit, are crucial to model the wireless environment.

Although extremely accurate, throughput prediction using mathematical models that fully capture the aforementioned channel effects are intractable when considering a higher number of connected devices, thus, they assume smaller deployments. Moreover, most of the existing mathematical models are based on assumptions (e.g., uniform traffic distribution and fixed transmission rate) that lower their complexity but, in turn, disregard the actual phenomena taking place in wireless networks. On the contrary, ML approaches can solve complex problems by learning from data, becoming a powerful network optimization tool. In fact, next-generation networks contemplate the upgrade of certain network functions by learning algorithms. Like the International Telecommunication Union (ITU), several standardization bodies have already defined an architecture where such data-driven ML models assist network communications.

As an example, the architecture proposed in [18] suggests using sandboxes, where multiple models can be trained with simulated data that closely resemble the underlying networking system. In this way, the robustness of ML models can be improved. Additionally, such frameworks also contemplate the use of an ML marketplace. Thus, an ML model is chosen depending on the capabilities and the contextual information that a WLAN can gather, e.g., node location and used data rate. Then, a simulation environment is prepared according to this information. The selected ML model is trained and evaluated in the sandbox or changed upon unwanted results. Once the evaluation is done, the ML model is deployed to the live networking system where the optimization takes place. This process implies that ML models are continuously learning about the wireless environment independently of the deployed technologies or services. For instance, to properly select a channel configuration, a throughput prediction model can be trained in a sandbox, using synthetic data from multiple deployments with multiple channel configurations to evaluate the network performance.

## 4. A GNN Model for Performance Prediction in Next-Generation WLANs

In several applications, data can be defined by graphs where the relationships among samples (or nodes) are represented by the edges between them. Networks are a clear example of such graphs; nodes have their own properties or features and relate to other nodes via the links between them. If there is no link between two nodes, we can generally say that those nodes are not related to each other. Despite their success, ML faces challenges when learning from structured data represented as graphs, as the relationships among nodes are not captured or have to be represented differently. For example, typical data pre-processing steps include the generation of a fixed-size matrix (e.g., images of a given size, audio samples or sentences of a fixed length), which represents the sample’s information and serves as training data.

**Figure 2:**
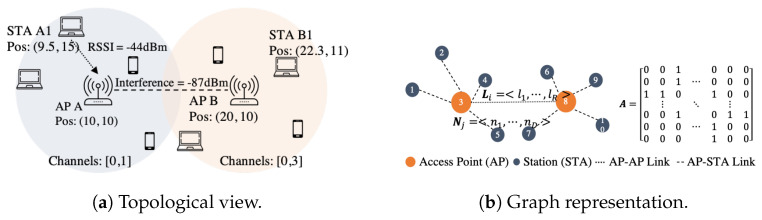
Example of a WLAN deployment. On the left, the topological view of the deployment, some measurements are included. On the right, the graph representation of the deployment with a node feature **N**_*j*_, an edge feature **L**_*i*_ and its adjacency matrix **A**.

Figure 2a shows a specific WLAN deployment of two BSSs (A and B). It consists of two APs, located at the center of the cell, and four STAs each, distributed around the coverage area of the APs (blue and orange circles, respectively). Information such as the position (x-y coordinate), channel configuration and the type of device, among others, can be used as features in a data set. To predict WLAN performance using a ML approach, a data set is created, using the aforementioned information, i.e., a vector represents the features of a device while a matrix represents a WLAN or a BSSs. Unfortunately, all deployments are different in terms of topology but also in terms of its size. By having a variable number of APs and/or associated STAs, the size of the matrix will also change, violating the requirement of ML of a fixed-size input. A ML practitioner could solve this issue by fixing the matrix size to the biggest deployment size on the data set and performing re-scaling or data padding on smaller deployments. Data padding can be seen as the introduction of new devices that do not alter the transmission pattern of the BSS, which is not totally true. Moreover, neural networks are usually trained in a fixed-sized batch-wise fashion where the size of the batch is determined by the capabilities of the computing machine. Normally, the bigger the size of the batch, the more computing power the machine should have. Typical values of batch size are 32 or 64 samples. This means that only 32 or 64 devices are considered per training batch, regardless of whether they belong to the same deployment or not.

On the contrary, approaches such as GNNs [19] have been proposed as neural networks that operate on graphs. Under GNNs, a graph is defined as a 2-tuple G=(V,E) with *V* representing a set of nodes with cardinality *N* and *E* representing the set of edges with cardinality *L*. Each node has associated a set of features, represented by a vector of 1×D, where *D* is the number of input features per node. Similarly, each edge represents the interactions among nodes; their associated features are represented by a vector of the form 1×R where *R* is the number of input features per edge. The graph’s topology is represented by a binary adjacency matrix of N×N, where a one represents an edge between nodes. Mapping the previous terminology to our specific example of Figure 2a, each deployment is considered as an undirected graph where STAs and APs are the graph’s nodes. Position, channel configuration and device type are considered to be the node features (more details in Section 6). The graph’s edges are established, according to the wireless interactions between nodes, e.g., the RSSI received by the AP and the interference levels between BSSs. In this case, Figure 2b shows the resulting graph of the deployment shown in Figure 2a. The graph has ten nodes (five per BSS) and nine wireless links, four per BSS plus one between APs. Once the data are formatted into a graph, there are several GNN frameworks (e.g., [20]) that operate on the node features, edge features and the adjacency matrices by creating sparse matrices.

For the sake of the argument, let us assume two WLAN deployments. The first deployment is shown in Figure 2 (called deployment *a*) while the second deployment (called deployment *b*) is an exact copy of the first. The adjacency matrix of deployment *a*, the node features and the target (throughput) are represented by matrix $A_{a}$, matrix $X_{a}$ and matrix $Y_{a}$, respectively. The same is valid for deployment *b*. Then, the GNN framework creates a new adjacency matrix, a new node feature matrix and a new target matrix as the concatenation of the two (*a* and *b*) around the node dimension as follows:

$$A=\left[\begin{array}{cc} A_{a} & \\ & A_{b} \end{array}\right],\;X=\left[\begin{array}{c} X_{a}\\ X_{b} \end{array}\right],\;Y=\left[\begin{array}{c} Y_{a}\\ Y_{b} \end{array}\right].$$

In this way, the operations (e.g., message passing) among two nodes of different deployments are not possible, and eliminates the padding in the node or edge features. Only the adjacency matrices are allowed to be padded, which has no change on the original topology (a zero in the adjacency matrix means that there is no edge between two pair of nodes).

To summarize, we advocate the use of GNNs in WLAN performance prediction, given the following:

- GNNs have been successfully applied to combinatorial optimization problems [21], and can achieve relational reasoning [22].
- CB is a problem with a combinatorial action space in dense deployments, where the complexity increases exponentially with the size of the deployment and the number of possible channel configurations.
- The relationships between STAs and APs (connectivity, interference, among others) can be captured via a graph representation, i.e., there is one-to-one mapping between the network topology and the graph representation.
- GNNs are also proposed to solve multiple network optimization processes [23], given their ability to generalize to large-scale problems.
- GNNs can easily operate and generalize over environments with a changing topology and a variable number of nodes.

We model a WLAN deployment as shown in Figure 2b. The links between STAs and APs represent the connectivity within the BSS through wireless links, while the link between APs represents the interference that BSSs have among themselves. As it can be inferred, APs form a mesh graph, while devices within the BSS form a star-like graph. We define our GNN model using Graph Convolutional Networks (GCNs) [24], a Convolutional Neural Network (CNN) variant operating in graphs. GCN follows a multilayer approach, just like FNN, with the difference that it processes graphs instead of images, time series, or text sequences. The number of Graph Convolutional Layers (GCLs) is typically defined by the diameter of the graph, which tells in how many hops any node is reachable from any other node; so, in this particular case, the graph diameter is three for inter-BSS interaction (two nodes of different BSS are communicating) and two for intra-BSS interaction (two nodes of the same BSS are communicating).

**Figure 3:**
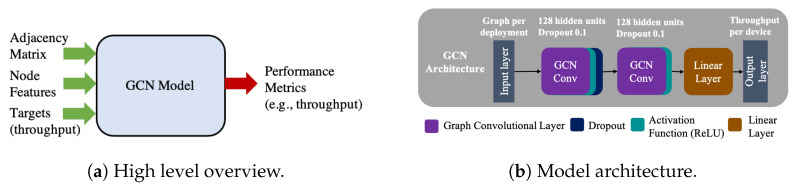
Summary of our GNN model. A high level overview is presented on the left, while the model architecture is presented on the right.

Despite GNNs being able to operate with edge features [22], GCNs do not naturally support them. As a consequence, all available features (detailed in Section 6) are defined as node features. Thus, the node features represent the deployment characteristics, such as node positioning, node type, channel configuration, and interference, among others. The deployments’ topology allows us to consider two GCLs to propagate information from STAs to AP and between APs. Additionally, as a third layer, we use a linear function that operates only on the node’s features. All GCLs use REctified Linear Unit (ReLU) as an activation function, and only the first GCL uses dropout for regularization purposes. Figure 3 shows a summary of our GNN model. Figure 3b shows the resulting architecture, with the best values found in a hyper-parameter search for the GCL. Figure 3a summarizes what our GCN model does; it takes as input a topology of WLAN deployment, represented in an adjacency matrix, the node features and the targets. It learns the wireless relationships between BSSs and predicts the performance of that WLAN deployment. Thanks to the generalization properties of GNNs, our model is able to predict the performance of unseen deployments, as shown in Section 7.

## 5. State-of-the-Art ML Models

**Figure 4:**
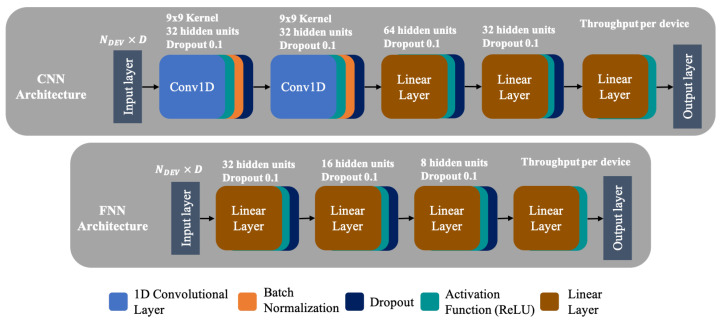
Architecture of the state-of-the-art ML models.

Here, we design two DL-based models and an ML-based model that serve as a baseline to our GNN model. Figure 4 (top and bottom) shows the DL-based model architectures. The input layer of all models receives the characteristics extracted from the deployment data. However, as each deployment does not have the same number of devices, the data have to be processed differently, as these models require a fixed size input. Here, data are pre-processed into a matrix of $N_{DEV}\times D$, where $N_{DEV}$ represents the number of devices in all WLAN deployments and is fed to the models using batches. In turn, the columns (*D*) represent the device’s considered characteristics (e.g., positioning, RSSI and set of bonded channels, among others). At the output layer, the throughput per device is predicted based solely on each device’s characteristics. This means that the pre-processing does not consider if a device belongs to a given deployment or not. We do not advocate for using data padding or resizing, as the variability in the deployment size is too big (see Section 6 for further details) and it would be wasteful in terms of computing resources, as most of the devices on a deployment would be missing.

As our GNN uses convolutional layers, we design a CNN that uses two 1D convolutional layers, followed by batch normalization and dropout layers to accelerate training and to reduce over-fitting, respectively. Thereafter, three linear layers followed by dropout layers are added. All layers have ReLU activation functions. Figure 4 (top) shows the resulting CNN architecture. The second model is a well-known FNN, shown in Figure 4 (bottom). It consists of four linear layers, the input and output layers, and two additional hidden layers. The number of neurons in the layers follows a decreasing configuration, i.e., 32-16-8-1. The rationale behind this configuration is that at each layer, the most representative data features are extracted. Each layer uses ReLU as activation function, followed by a dropout layer to reduce over-fitting. Lastly, the predicted throughput is obtained at the output layer, configuring a regression model.

The last model is based on Gradient Boost (GB). Boosting algorithms are ensemble methods that minimize the model’s loss by adding decision trees, using a gradient descent procedure. In this method, we use default hyper-parameter values, defined by sci-kit learn (https://scikit-learn.org/stable/modules/generated/sklearn.ensemble.GradientBoostingRegressor.html (accessed on 21 June 2021)). FNN and GB models are typically used in Wi-Fi performance prediction [13,14].

## 6. Data Set

### 6.1. Simulated Data Sets for Training Ml-Based Networking Solutions

Typically, current available Wi-Fi data sets (https://data.europa.eu/data/datasets/jrc-netbravo-netbravo-od-eu-wifi?locale=en (accessed on 21 June 2021)) do not include the novel CB feature nor represent dense deployments. Working with data sets with sparsity (e.g., deployments with a limited number of transmitters and receivers) prevents finding insights on next-generation high-density deployments, where the severity of the interference is expected to generate a great impact on the performance. Therefore, to train the models presented in Section 4 and Section 5, we used the dataset in [25], which was provided in the context of the 2020 edition of the *ITU AI/ML for 5G Challenge* (https://www.itu.int/en/ITU-T/AI/challenge/2020/Pages/default.aspx (accessed on 21 June 2021)), where our team ATARI was awarded with the 4th position in the Grand Challenge Finale among 33 finalists selected from 911 teams competing in 23 open challenges. This data set includes simulated data from IEEE 802.11 WLAN deployments applying DCB. More specifically, the simulated data set was generated using Komondor [26], an IEEE 802.11ax-oriented open-source network simulator that was conceived as a cost-effective simulation tool for studying the performance of novel features, such as DCB or Spatial Reuse (SR) in dense scenarios. As shown in [8], important features in Komondor, such as the Distributed Coordination Function (DCF) operation and DCB, were validated against ns-3 [27] and the well-known Bianchi and Markov models [6,28].

Moreover, the simulator was already used in real-world testbeds [7]. In this case, Komondor characterized the WLAN testbed and generated a simulated network twin. An ML approach for transmit power control was trained, using the data generated by Komondor, and then it was applied to the real testbed. The results showed that in using the ML approach, the real deployed WLANs experienced an increase of at least 76% in its throughput.

Unlike the existing measurement campaigns of DCB WLANs (e.g., [29]), Komondor provides synthetic traces depicting different types of deployments, ranging from low to ultra-high density. As shown in [29] through real traces, the 5 GHz band used for DCB in 11ax and 11be amendments is deeply underutilized, even in highly populated areas. Accordingly, network simulators can contribute to filling the data gap in next-generation network deployments and, therefore, provide comprehensive data of interactions among devices in future deployments at which channel occupancy is expected to increase significantly. The broad range of situations captured with large simulated datasets can be very useful to train data-hungry ML models, such as CNNs or FNNs.

The role of network simulators in future 5G/6G networks has been pointed out as a key tool to assist the increasingly adopted ML operation in communications [7]. The fact is that network simulators can represent unknown situations that may not be present in real traces (due to the limitations in acquiring data from real networks), thus allowing to support procedures, such as training, testing, and validation of ML models.

### 6.2. Data Set Generation

The data set is divided into two sets, i.e., training and testing. In both cases, enterprise-like scenarios containing a different number of APs and STAs applying DCB are generated, thus depicting multiple situations that could be used for training ML models. The topology of these enterprise-like scenarios is composed of a building floor of a given size (e.g., map size), which is divided in equal-sized offices. The APs’ positioning is fixed, while the STAs are randomly placed around the AP’s coverage area. Such a topology setting is typically adopted in the simulation scenarios provided by IEEE 802.11 task groups [30]. The data set includes useful information about each deployment, such as the obtained throughput, the RSSI, the airtime in each channel, the interference among devices, or the SINR.

In total, 600 deployments were simulated, containing 78,078 devices (6000 APs and 72,078 STAs). Simulating each CB deployment for training and testing took in the order of tens and hundreds of seconds, depending on the deployment features (e.g., number of nodes, traffic load, and packet losses). Table 1 summarizes the main characteristics of the entire data set. Moreover, Table 2 details the simulation parameters used for generating the data sets. For more details regarding the parameters used to characterize 11ax frames, please refer to [26]. Notice, as well, that 100 and 50 random deployments were simulated for each type of scenario in training and test data sets, respectively. In all the cases, 10 s simulations were considered. (There is a trade-off between the simulation time and the stability of the simulated results. In particular, 10 seconds was shown to properly address this trade-off by providing accurate results at a low execution time in Komondor [7].).

Regarding the DCB configuration, each BSS can use up to N=8 basic non-overlapping channels of 20 MHz in the GHz band. Compliant with the 11ax amendment, a given transmitter can bond channels of width 20 MHz, 40 MHz, 80 MHz, and 160 MHz, thus leading to channelization C={{1},{2},⋯,{8},{1−2},{3−4},⋯,{7−8},{1−4},{5−8},{1−8}}, for basic channels indexed from 1 to 8 (see Figure 1a). In each simulated deployment of the data set, both the primary and the total channel width are selected randomly. As for the applied DCB policy, the maximum possible channel width is dynamically used, provided that the involved channels were free during at least the point coordination function interframe space (PIFS) period. For instance, let us assume that a given transmitter has randomly allocated to channels {1−4}, with primary channel 1. Then, such a device would perform carrier sensing in the primary channel (1) and, provided that the channel was sensed to be free during the backoff, would assess whether the rest of the channels were also found to be free during the PIFS interval. If only channels {1−2} are idle at the moment of starting a transmission, then the transmitter proceeds to use both of them, leaving channels {3−4} for future transmissions.

**Figure 5:**
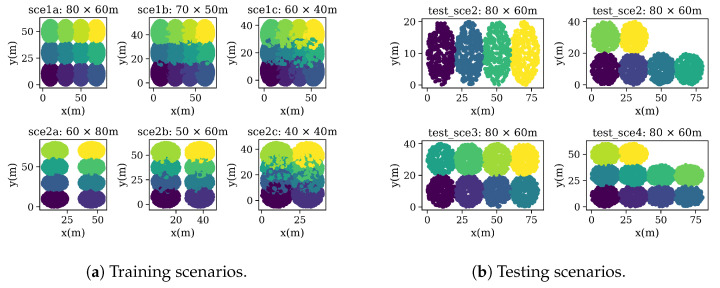
Spatial distribution per training and testing scenarios. Different colors represent the coverage area of a BSS. APs are located in the center of the circle.

Having multiple random deployments with several APs and STAs, using different DCB configurations, motivates the use of GNNs. The fact is that GNNs exploit the graphs’ topological information, independently of how many nodes the graph has, by aggregating neighboring nodes’ information. Moreover, each scenario introduces a given amount of interference among BSS, as shown in the spatial distribution in Figure 5a. For instance, training scenarios *1a* and *2a* only consider inter-STA interference, while training scenarios *1c* and *2c* consider overlapping among BSSs. In the former (e.g., *1a* and *2a*), phenomena such as collisions by hidden-node are prone to occur due to the downlink traffic profile and given the fact that APs do not sense each other. Moreover, for the latter case (e.g., *1c* and *2c*), flow starvation can occur if two or more APs monopolize the channel, thus making other APs contend for the channel excessively. With DCB, this situation may be generated by a chain effect, given that APs select the transmission channels, according to their utilization.

### 6.3. Data Set Analysis

In the pre-processing step, the relevant data were extracted and appropriately formatted for later processing at the ML approaches. Based on Shannon’s theorem of channel capacity, we defined two extra features: the distance between AP and its associated STA; and the bandwidth of an AP, defined as the number of bonded channels multiplied by the bandwidth of an individual channel (20 MHz). We also noticed that the *Primary Channel, Minimum*, and *Maximum Allowed Channels* define a configuration of wireless channels. Therefore, these features are one-hot encoded into one categorical variable (*Channel Configuration*), representing the set of channels that a node uses. We identified that from fifteen possible channel configurations (see Figure 1a), only six channel configurations (from C0 to C5) were used in training and testing data sets. From those, configurations C4 and C5 were mostly used in both data sets. Table 3 summarizes the features used during training.

**Figure 6:**
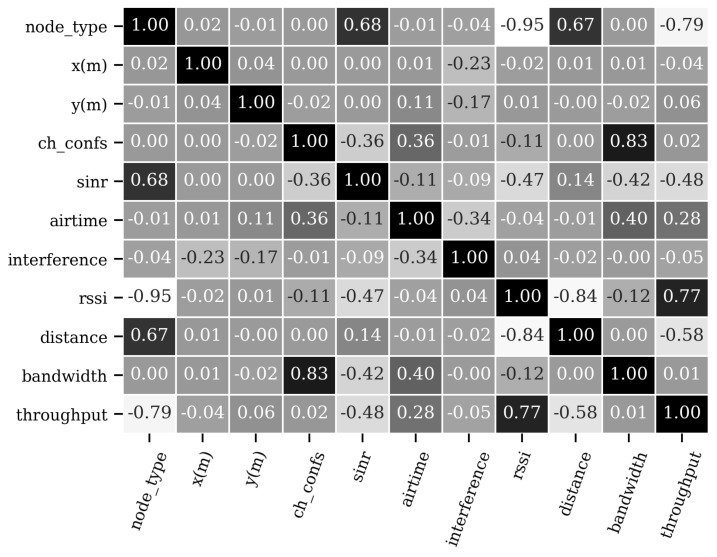
Correlation between features and throughput.

We performed a correlation analysis on the training data set to see which of the defined features (independent variables) has more impact on throughput prediction (output variable). Figure 6 shows the correlation matrix, using the Pearson correlation. A value close to one (or negative one) shows a strong (negative) correlation, while a value close to zero implies no correlation between variables. It is worth to mention that this analysis was not carried out on the raw data, which include several undefined values (e.g., RSSI is set to *∞* for APs), but it was already pre-processed and ready to serve as input to the different models. For instance, distance is not correlated with the x-y coordinate, as it is measured from the AP to the STA; hence, the distance of APs is always set to zero. Moreover, due to interference phenomena, it is not clear that RSSI, SINR and distance keep a linear relationship with the throughput.

As can be seen, features such as node type, SINR, airtime, RSSI, and distance are more relevant for correctly predicting the throughput. Good signal strength is one prerequisite for higher performance, but it is not the only factor determining it. In wireless transmissions, the strength of interfering transmissions is also important to decide which channels are bonded. Moreover, when contending for a transmission slot, the more airtime is given to an STA, the higher the amount of data it transmits. Hence, there is a positive correlation between the airtime and the throughput. The correlation matrix provides a good starting point for feature selection, as features with correlation values closer to zero can be disregarded. However, if two variables are highly correlated, they may add noise to predicting the output variable, negatively impacting the model’s performance.

**Figure 7:**
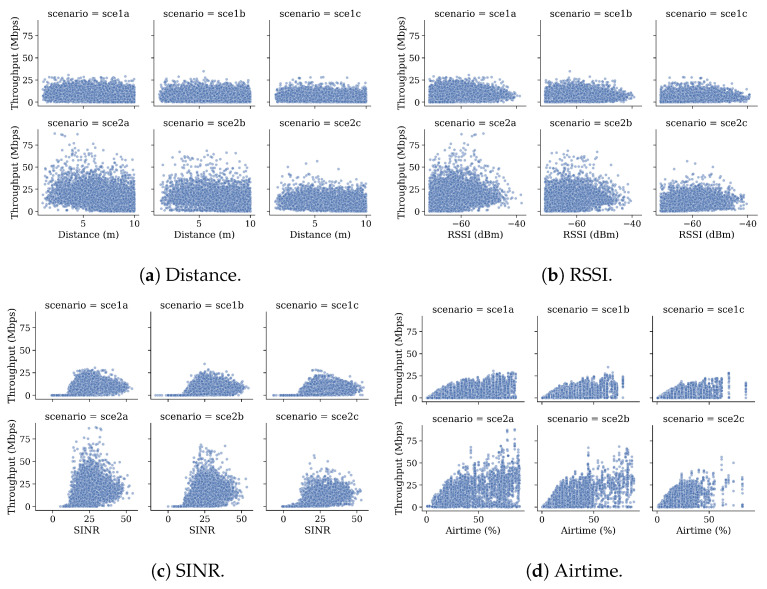
Relationship between throughput and main features per training scenario.

Figure 7 graphically shows the relationship between the most relevant continuous variables and throughput per scenario. It is shown that, in general, scenarios *2a*, *2b*, and *2c* present higher throughput than scenarios *1a*, *1b*, and *1c*, regardless of the value of other variables, showing the problem’s severity in highly dense scenarios. Figure 7a shows that STAs near the APs typically have better throughput in less crowded scenarios. However, this condition does not hold in more crowded scenarios, where the throughput is more or less similar for all the STAs. Regarding the RSSI, Figure 7b shows that it is not linearly related to throughput. Therefore, higher RSSI values do not always represent higher throughput. In fact, higher throughput values are found around −60 dBm, which confirms that indeed, RSSI alone is not a good indicator of an adequate link quality [32]. Similarly, in Figure 7c, higher throughput values are found at mean values of SINR. A more direct relationship can be found in Figure 7d, where generally, higher values of airtime are associated with higher values of throughput.

Based on these conclusions and the correlation matrix, we defined several experiments where different features are used to train the models, and are summarized in Table 3. The experiment selection was also motivated because features such as node type and RSSI can be easily gathered in currently available WLANs. By contrast, features such as distance or airtime are more difficult to obtain. For instance, experiment 2 (E2) uses the most relevant features found during data exploration, namely, node type, SINR, airtime, RSSI, and distance. The remaining experiments are defined by taking one of the most relevant features (e.g., SINR in E5) and its uncorrelated features (e.g., node position and interference), while its counterpart (E6) tries to measure the relevant feature’s impact by repeating the same experiment (E5) without including it.

## 7. Results

This section summarizes the obtained results and the comparison between the proposed models. We also provide insights into the impact of different features in the models’ performance on throughput prediction.

### 7.1. Training and Validation

The models described in Section 4 and Section 5 were trained on the corresponding data set with a fixed split (80% for training and 20% for validation). In our GNN approach, we considered each deployment as a graph, which means that 480 graphs were used for training and 120 for validation. Every model uses the Root Mean-Squared Error (RMSE) as a loss function, defined below. The error was obtained across the predictions (${\hat{x}}_{i}$) compared to the actual results ($x_{i}$), where *N* is the number of devices in the batch. Note that the batch size for the GNN model is 32 graphs, while traditional DL approaches use a batch size of 32 and 128 devices for the FNN and the CNN, respectively.

The used data set considers several deployments in similar settings (e.g., fixed 12-AP setting in scenarios *1a*, *1b*, and *1c*. See Table 1), which allows the ML approaches to finding patterns in the data to build a general representation of the network. This representation does not perfectly fit every single training datapoint because otherwise, the models will overfit. On the contrary, this representation is the one that minimizes the error between the datapoint and the prediction.

$$RMSE=\sqrt{\frac{\sum_{i=1}^{N}{\left(x_{i}-{\hat{x}}_{i}\right)}^{2}}{N}}$$

However, during data analysis, we found that the mean STA’s throughput is much lower than the mean AP’s throughput (see Table 1). Therefore, the throughput of the APs is considered an outlier for any ML approach. Additionally, the initial exploratory results showed that the models are mostly focused on correctly predicting the throughput of the APs, given that the RMSE is minimized on large values. Consequently, we proposed a masked loss where only the STAs contributes to the loss. In other words, the network does not need to learn to predict the AP throughput. The AP’s predicted throughput is computed at a post-processing step by summing its associated STAs’ predicted throughput. The RMSE is calculated, using the STAs’ predicted and the APs’ computed throughput.

The model’s (FNN, CNN, and GNN) hyper-parameters were selected, using a hyper-parameter search over hundreds of executions. This search’s best results were obtained, using Adam optimizer with a learning rate of 0.001. The models were implemented in Python, using Tensorflow 2.1.2 for FNN and CNN and PyTorch Geometric [20], a geometric deep learning extension library for GNN. The training was accelerated by using GeForce GTX 1080 Ti GPUs in our GPULab facility (https://gpulab.ilabt.imec.be (accessed on 21 June 2021)). We trained the models on each set of features defined in Table 3 to quantify how they affect the prediction accuracy. This procedure was performed ten times per experiment to observe each model’s convergence.

### 7.2. Performance Evaluation of the Proposed Models

The testing data set also includes several deployments in enterprise-like scenarios with same channel configurations. However, changes regarding the training data set, including map size, deployment size, and each deployment’s user density, were introduced and are shown in Figure 5b. In this case, four scenarios were simulated, according to the number of considered APs in a fixed map size (see Table 1).

As a baseline for all ML approaches, we use a random guesser. We assume that the throughput can be obtained from a normal distribution with the mean and standard deviation found during data analysis to build this random guesser. Given that the throughput in STAs varies between 0 and 88 Mbps, we use a truncated normal distribution between those values. This random approach represents a naive and cheap way to generate predictions in this particular problem.

**Figure 8:**
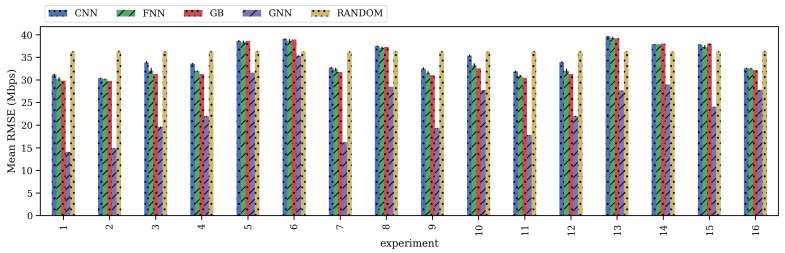
Mean and standard deviation of the obtained RMSE by all models on the test data set.

The trained models were used to predict the throughput of all devices in the test data set. Figure 8 shows the mean RMSE across all test scenarios’ deployments in a given experiment for all the generated models and its standard deviation. The standard deviation in all models is very low, having a maximum of 0.66 in the FNN. As can be seen from the figure, GNN outperforms all other approaches in all defined experiments. The random approach performs more or less the same, independent of the features used. Regarding the other ML models, it can be seen that learning from data represents at least a 20% improvement regarding the random approach, using all trainable features (E1). Focused on E1, i.e., the experiments with all features, GNN can obtain up to 64%, 56%, 55%, and 54% when comparing it against the random approach, the CNN, the FNN, and the GB, respectively. Surprisingly, GB performs slightly better in several experiments when compared to the CNN and the FNN. Despite its complexity, the CNN does not perform better than the FNN and the GB. This poor performance might be due to data representation. CNNs outperform other ML approaches when dealing with high-dimensional data (e.g., images, time-series). Even though the wireless environment is too complex to be modeled, the provided data do not include an extra dimension (e.g., time) that CNN can benefit from.

In some experiments (e.g., E5, E6, E8, E13, E14, and E15), the random guesser performs better than some ML approaches since the combination of input features does not benefit from the learning process. In fact, the Gaussian assumption works well in some cases and it is widely adopted in many communications aspects. Moreover, the random guesser performs more or less the same, independent of the input features, while for some experiments, the ML approaches benefit from certain features (e.g., E1, E2). This random guesser includes some basic information and performs better than a best-effort approach. It serves as an upper bound to the ML approaches, as it shows if the ML model is actually learning.

In terms of feature relevance, including airtime, there is a strong improvement to the results of all ML models. For instance, the only difference between E1/E7 and E15/E8 is that the latter does not consider airtime while the former does. It can be seen that not considering airtime represents between 85% and 87% decrease for the GNN, while other ML approaches perform even worse than the random approach. Nonetheless, considering only airtime as an input feature does not ensure good performance. For example, in E16, GNN decreases its performance by more than 100% regarding E1, except for other ML approaches, where using airtime as an input feature performs even better than considering the rest of the features (see E15).

Analyzing other features, RSSI and distance give more information about the throughput than SINR, node type, and interference. For instance, in E9 and E10, all models improve their performance by including RSSI: 48%, 5%, 10%, and 6% in the GNN, GB, CNN, and FNN, respectively. Similarly, in E11 and E12, GNN obtains around 26% improvement, while CNN and GB obtain 2.4% and 7.5% improvement, respectively, when considering the distance. Interestingly, the GNN is the only model in which features such as node type (E3 vs. E4) and SINR (E5 vs. E6) are relevant and improve its performance. Even when considering interference (E13 vs. E14), a factor that seems to decrease other ML models’ performance, GNN obtains a 5% improvement.

Based on our experiments, we observe a low correlation between the location of APs and the performance of STAs. Some experiments use the devices’ location for training the models (e.g., E1), but this information is not used in other experiments (e.g., E2). There is a slight drop in accuracy between E1 and E2 but, in general, the position of the APs does not impact greatly on the performance of the models. This conclusion is also motivated by the correlation matrix shown in Figure 6 that suggests that the positioning of the nodes (x and y features) is not a relevant feature for determining the throughput. In turn, other features are more representative and might encode the positioning of the nodes (e.g., SINR, distance, and RSSI).

**Figure 9:**
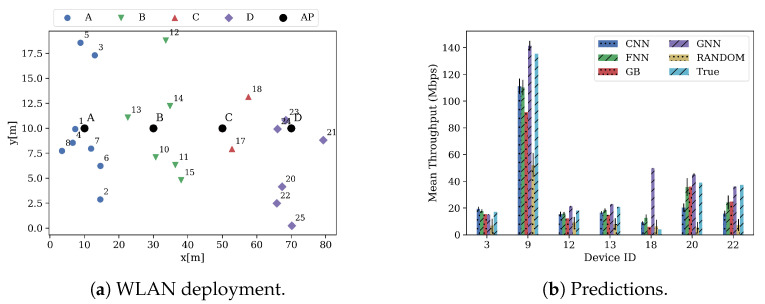
Deployment and predictions of some devices of test scenario 1.

Zooming in on the quality of the predictions, Figure 9 shows a random deployment of the scenario *1* in the test data set (Figure 9a), and the mean predicted throughput by all the models (Figure 9b) in E1. For visualization purposes, only seven devices per deployment were randomly selected. As explained in the previous section, APs’ throughput is relatively higher than STAs’ throughput since it is aggregated from its associated STAs. This can be seen in device 9, which represents the AP *B* (see Figure 9a), where it aggregates the throughput of STAs 12 and 13. As it can be seen, all the models accurately predict the expected throughput of the BSS. However, GNN predictions are, in general, less accurate than the predictions performed by other models (excepting the random guesser). In less crowded scenarios, GNN presents more difficulties in predicting the significantly lower STAs’ throughput (device 18). On the other hand, GNN is better than other approaches at predicting large throughput values (devices 9, 20 and 22).

**Figure 10:**
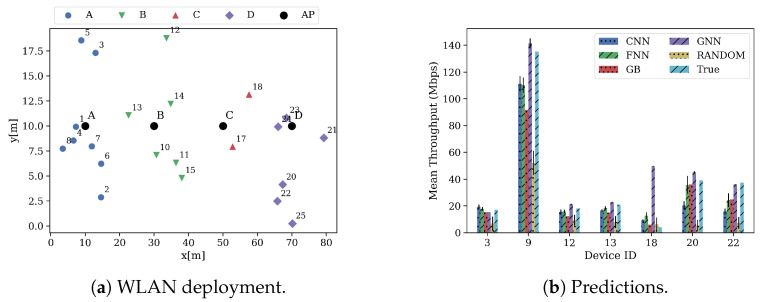
Deployment and predictions of some devices of test scenario 4.

Focusing on more crowded scenarios, Figure 10 shows a random deployment of the scenario *4* in the test data set in E1. Figure 10a shows the deployment, while Figure 10b shows the predictions. In this case, GNN manages to accurately predict smaller throughput values (devices 4, 5 and 42) yet, it presents difficulties at predicting larger throughput values (devices 12 and 44). Nonetheless, its predictions are significantly better than the ones made by the other models in these same devices.

Note that the RMSE in Figure 8 is lower than the one that can be inferred from Figure 9b and Figure 10b; because it is computed using all the predictions from all scenarios’ deployments and from all runs, i.e., the considered number of devices, *N* is larger than the number of devices in a deployment.

In general, the accuracy of the ML models strongly depends on the features used for training them. Therefore, based on the available features, a management system might select a given model from the marketplace, sacrificing model accuracy. The results also showed that approaches such as GNN benefit from having more features, as the model obtained better performance in each performed experiment that included a given feature versus its complement. Nonetheless, GNN was able to outperform other ML models, independent of the used features, given its ability for learning relationships among nodes in a graph.

## 8. Discussion

The development of the 5G era has exacerbated the network complexity due to several reasons. One of them is the shift of the paradigm toward programmable networks that can be holistically orchestrated to offer end-to-end services. This network programmability is accompanied by an extreme set of requirements, such as ultra-low latency, highly reliable communication, and improved user experience. Therefore, to address this large set of requirements and diversity of network services, networks should be fully automated to accelerate service delivery while meeting economical goals.

However, current network architectures are unable to offer a high degree of automation, and network providers still rely on manual configuration. Network models and optimization algorithms are developed for that purpose. In this way, network administrators set an intent [33,34] as input to the optimization algorithm, and through the model, the algorithm finds the network configuration that fulfills the requirements. For instance, in the particular case of CB in IEEE 802.11 (Wi-Fi) networks, traditional Markov models and other mathematical models have been developed to predict the performance (e.g., expected throughput) WLANs in the presence of interference, given a deployment (e.g., topology) and a particular configuration (e.g., set of channels that could be bonded). As discussed in Section 2, these models are based on simplifications and are limited by smaller deployments in order to keep feasibility.

**Figure 11:**
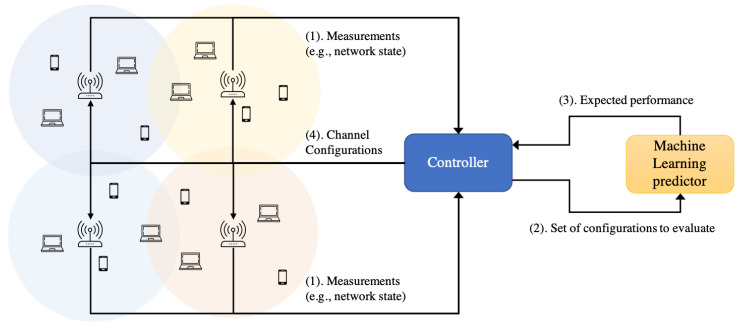
Usage of ML predictors for network optimization.

NNs are known to be good function approximators [35]. By learning from data, NNs are able to build a function that abstract complex network behavior. In the previous sections, we trained and validated several ML models to predict the expected throughput of a deployment of different WLANs under CB with the aim to offer optimization algorithms a reasonable network model that accurately explores the performance of possible channel configurations as shown in Figure 11.

Firstly, different measurements are constantly collected from the network and delivered to the controller. The collected measurements constitute the network state. The measuring time depends on the kind of decision that is being optimized. For instance, long-term metrics, such as energy consumption, do not require short monitoring intervals. Although some of the features are difficult to get, we expect that the introduction of Software Defined Networking (SDN) in wireless networks will facilitate the data collection campaigns, where the collected data can later be used for intelligent management decisions.

Then, the evaluation of different configurations will be executed in a timely manner if a triggering condition is met, forming a closed control-loop, for example, if the controller perceives that 50% of their managed devices have lower throughput than expected. In this case, the ML model will generate a prediction for every evaluated configuration. Note that a traditional model (e.g., Markov model) takes a long time to generate a prediction, longer than the time required to optimize the spectrum usage to decrease interference and improve the user experience.

Undoubtedly, the role of the controller is vital for network optimization, as it is in charge of making the management decisions. Nonetheless, an accurate and fast network model is also a key element within the architecture. In this paper, we addressed the latter and leave the controller out of the scope. In particular, we presented, trained and evaluated a GNN model that is well suited to the WLAN performance prediction problem, where information is also embedded in the topological representation. We believe that the combination of powerful data-driven models as controllers and predictors enables the vision of self-organized, self-monitored and self-healing beyond 5G networked communication.

It is clear that devices not participating on the proposed system may affect the performance of the same. However, if the channel configuration of the devices is known (e.g., devices with a fixed configuration, not using DCB), this information can be included in the generated data set in all its deployments. In this way, the fixed configuration is learned by the model to improve its prediction.

## 9. Conclusions

Future wireless networks will be highly dense and will require higher performance than what we have today. To address such an increasing complexity, Artificial Intelligence (AI)/ML plays an essential role in modeling and driving wireless networks’ behavior. In this paper, we focus on the performance prediction problem for next-generation WLANs, applying CB techniques, which underlying complexity has, until now, hindered the utilization of analytical models.

For that purpose, we propose a GNN model that adapts well in graph-based problems that exhibit combinatorial behavior. Additionally, we compared our GNN model to more traditional ML models and analyzed the impact of different features on the model’s performance. Depending on the available data, a controller might use a trained model with a given set of features or another. According to the evaluation, our GNN approach can obtain a 64% increase in the performance regarding a naive approach and around 55% for other ML approaches when using all training features.

Several challenges are still open and can be addressed in future work. For instance, the generated data considered that two channels could be bound, at the least. To complete the study, it is worth including legacy WLANs that can only transmit over one unbonded channel. Secondly, more dynamic scenarios can be considered if several “snapshots” of the network status can be taken to exploit the spatio-temporal dependence of the link quality with the achieved throughput, allowing models such as CNN to perform better.

Additionally, in this work, we only considered Wi-Fi interference. However, given the wide range of technologies that are accessing the spectrum (e.g., ZigBee and Bluetooth), it is important to evaluate the impact of non-Wi-Fi interference on the models. The fact is that non-Wi-Fi signals are treated differently than Wi-Fi ones (Channel Clear Assessment (CCA) vs. Energy Detection (ED)), so there would definitely be an impact on the simulation results. For instance, features such as SINR will reflect a degradation, as the higher the interference caused by other technology, the lower the SINR and, correspondingly, the lower the throughput. As a result, the accuracy of the model is tied to the ability of the same for differentiating between those different types of interactions. For instance, if the model is able to differentiate whether a contention is produced by a Wi-Fi or a non-Wi-Fi signal, that would contribute to provide more accurate predictions on CB configurations suitability.

Despite the provided data set including the basic 11ax CB operation with contiguous channels, both the data set and this work constitute an initial step for large-scale modeling of WLANs applying DCB by using ML. However, as future wireless networks will most likely be based on OFDMA, future work should include such a type of operation in its evaluation. As stated in Section 3, the direct consequence of including OFDMA operation in DCB is the increase in the number of available channel configurations since non-contiguous channels could be bonded. In this way, ML approaches will become even more meaningful for this type of WLAN modeling, given that traditional approaches will fail in obtaining an accurate prediction of the WLAN performance, considering large-size networks and the combinatorial search space.

## Figures and Tables

**Table 1:** Summary of data set characteristics.

Data Set	Scenarios	Map Size	№ Deployments	Total Devices	APs Per Deployment	STAs Per Deployment	Mean–Std–Min–Max STA Throughput	Mean–Std–Min–Max AP Throughput
Training and Validation	1a	80 × 60 m	100	78,078: 6000 APs 72,078 STAs	12	[10–20]	[6.93–6.99–0–88] Mbps	[83.29–52.24–0–400] Mbps
	1b	70 × 50 m	100		12	[10–20]		
	1c	60 × 40 m	100		12	[10–20]		
	2a	60 × 40 m	100		8	[5–10]		
	2b	50 × 30 m	100		8	[5–10]		
	2c	40 × 20 m	100		8	[5–10]		
Testing	1	80 × 60 m	50	9831: 1400 APs 8431 STAs	4	Random	N/A	N/A
	2	80 × 60 m	50		6	Random		
	3	80 × 60 m	50		8	Random		
	4	80 × 60 m	50		10	Random		

**Table 2:** Simulation parameters used to generate the training and test datasets.

	Parameter	Value	
		*Training*	*Test*
Depl.	# APs	{8, 12}	{4, 6, 8, 10}
	APs location	Fixed to the center of the cell	
	# STAs	{5–10, 10–20}	5–10
	STAs location	Uniform at random	
	Traffic profile	Downlink UDP	
	Traffic load	Full buffer mode	
	Channel allocation	Uniform at random	
PHY	Central freq.	5 GHz	
	Path-loss model	See [31]	
	Bandwidth	{20, 40, 80, 160} MHz	
	# spatial streams	1	
	Allowed MCS indexes	1–12	
MAC	Contention window	32 (fixed)	
	Data and ACK length	12,000/32 bits	
	RTS and CTC length	160/112 bits	
	Max. A-MPDU	1	
	DCB policy	Dynamic (see [12])	

**Table 3:** Features used per experiment.

Feature	Definition	E1	E2	E3	E4	E5	E6	E7	E8	E9	E10	E11	E12	E13	E14	E15	E16
Node Type	Wireless node type, AP = 0, STA = 1	✓	✓	✓				✓	✓					✓	✓	✓	
x(m)	x-coordinate of the wireless node	✓		✓	✓	✓	✓	✓	✓	✓	✓	✓	✓			✓	
y(m)	y-coordinate of the wireless node	✓		✓	✓	✓	✓	✓	✓	✓	✓	✓	✓			✓	
Channel Configuration	Combination of Primary, minimum and maximum channel	✓		✓	✓							✓	✓	✓	✓	✓	
SINR	Signal to Interference plus Noise Ratio	✓	✓			✓										✓	
Airtime	Percentage of time each AP occupies each of the assigned channels (mean)	✓	✓	✓	✓			✓		✓	✓	✓	✓				✓
Interference	Inter-AP interference sensed from every AP (mean)	✓		✓	✓	✓	✓			✓	✓	✓	✓	✓		✓	
RSSI	Received Signal Strength Indicator	✓	✓					✓	✓	✓						✓	
Distance	Euclidean distance between AP and STAs	✓	✓					✓	✓			✓				✓	
Bandwidth	Maximum channel bandwidth	✓		✓	✓							✓	✓	✓	✓	✓	

## Data Availability

The data set used to conduct this study is open-source and accessible at http://doi.org/10.5281/zenodo.4106127 (accessed on 21 June 2021).
